# Practical limitations of monocyte subset repartitioning by multiparametric flow cytometry in chronic myelomonocytic leukemia

**DOI:** 10.1038/s41408-019-0231-7

**Published:** 2019-08-16

**Authors:** Prateek A. Pophali, Michael M. Timm, Abhishek A. Mangaonkar, Min Shi, Kaaren Reichard, Ayalew Tefferi, Kevin Pavelko, Jose C. Villasboas, Dragan Jevremovic, Mrinal M. Patnaik

**Affiliations:** 1 Division of Hematology, Department of Internal Medicine, Mayo Clinic, Rochester, MN USA; 2 Department of Laboratory Medicine and Pathology, Mayo Clinic, Rochester, MN USA; 3 Department of Immunology, Immune Monitoring Core, Mayo Clinic, Rochester, MN USA

**Keywords:** Myelodysplastic syndrome, Translational research

Dear Editor,

Monocyte subset repartitioning by multiparametric flow cytometry has recently been shown to be an effective tool in delineating patients with chronic myelomonocytic leukemia (CMML), from other reactive and clonal causes of monocytosis^1–3^. Based on the expression of CD14 and CD16, monocytes can be divided into three categories; CD14^+^/CD16^−^ classical (MO1), CD14^low^/CD16^+^ intermediate (MO2), and CD14^−^/CD16^+^ non-classical monocytes (MO3), respectively^1,2^. These subsets differ in their chemokine receptor expression, phagocytic activity, gene promotor/enhancer profiles and have unique metabolic pathway dependencies^2,4^. It has also been shown that downregulation of hsa-miR-150 through methylation of lineage-specific promotors in CMML monocytes, results in impaired differentiation of MO1–MO3 monocytes, with *TET3* being a potential target^5^.

In the pivotal French study, monocyte subset profiling by flow cytometry in patients with CMML, consistently demonstrated an increase in the MO1 subset, with an established cut off of >94% being associated with sensitivity and specificity values of 90.6% and 95.1%, respectively^2^. These findings were validated by independent groups and importantly this method was found to be effective in identifying patients with myelodysplastic syndromes (MDS) that eventually evolved into CMML and in distinguishing CMML from patients with myeloproliferative neoplasms (MPN) with monocytosis^1,3,6^. That being said, the uniform adaptability of this test in routine clinical practice has important limitations, with false negative findings secondary to autoimmunity/inflammation (expansion of the MO2 fraction) and false positive findings in related myeloid diseases, such as MDS and atypical chronic myeloid leukemia a(CML)^3,7^. The impact of prior Hypomethylating agent (HMA) therapy and individual somatic mutational profiles also needs elucidation. A recent study has suggested higher sensitivity and specificity values for CMML detection using a MO3 cut off of <1.13%^7^. We carried out this study to (i) ascertain the utility of monocyte subset analysis by flow cytometry in patients with myeloid malignancies with monocytosis, (ii) assess the sensitivity and specificity of MO1 and MO3 cut offs in newly diagnosed CMML patients, and (iii) describe false positive cases.

We included 113 patients with myeloid neoplasms including 43 with CMML, 29 with MDS, 20 with MPN, 16 with MDS/MPN unclassified, and five with CML as defined by the 2016 WHO criteria^8^, along with 71 controls with reactive monocytosis (absolute monocyte count/AMC > 1 × 10^9^/L). Diagnostic bone marrow (BM) biopsies were reviewed independently by pathologists. Peripheral blood (PB) samples were subjected to flow cytometry by using the following methodology; whole blood (100 μl) was placed in a 12 × 75 falcon tube and washed once in 3 ml of PBS to remove soluble CD16. Cells were then stained with antibodies to CD3 V450, CD7 BB515, CD14 APC H7, CD16 Percp Cy5.5, CD33 PE Cy7, CD45 APC, CD56 APC R700 (BD biosciences, San Jose, CA) and CD24 PE(Biolegend, San Diego, CA). Red Blood cells were then lysed with 2 ml BD Facs Lyse (BD biosciences, San Jose, CA), washed, and re-suspended in PBS. Monocyte subsets were identified using Kaluza Software (Beckman Coulter, Brea, CA). A liberal ssc/CD45 gate was set where monocytes typically reside. Other lineages were excluded using antibody combinations in the panel; T cells were excluded with CD3, B-cells and granulocytes with CD24, NK cells with CD7 and CD56. The purified monocytes were then compartmentalized into the MO1, MO2, and MO3 subsets based on their CD14 and CD16 expression^9^.

Mass cytometry was also carried out on a select group of CMML PB samples (*n* = 5) and controls (*n* = 5). Single-cell suspensions were stained with a cocktail of metal-tagged antibodies recognizing 29 surface proteins (Supplementary Table 1). Nucleated cellular events were identified using a DNA intercalator conjugated to natural abundance iridium (191Ir and 193Ir). Cisplatin (195Pt) was used for dead-live cell discrimination and calibration beads containing natural abundance cerium (140/142Ce), europium (151/153Eu), holmium (165Ho), and lutetium (175/176Lu) were used for normalization of instrument signal within experiments. Our panel was constructed using commercially available metal-conjugated antibodies (Fluidigm Corporation) and stained cells were acquired on the Helios mass cytometer (Fluidigm Corporation). Data processing was performed using GemStone (Verity Software House) which used probability state modeling to assign cells to canonical subsets.

All but three (4.2%) patients with reactive monocytosis (*n* = 71, median AMC 1.7 × 10^9^/L) had MO1 fractions <94% (one with an IgG kappa monoclonal gammopathy, one with metastatic lung cancer, and one after aortic valve surgery), while eight (11%) patients had M03 fractions <1.13% (none with an underlying malignancy). At last follow up (median 23 months), none of these patients had evolved into a myeloid neoplasm. We then assessed the ability of this assay to help differentiate CMML from myeloid malignancies with absolute monocytosis and only included patients naïve to epigenetic therapies/HMA, *n* = 16; MDS-1, MPN-5, MDS/MPN-U-8, and CML-2. All five patients with MPN with monocytosis (primary myelofibrosis–3, polycythemia vera–1, and *CSF3R* mutated chronic neutrophilic leukemia–1) had MO1 fractions of <94%, while two (40%) had MO3 fractions <1.13%. Among two patients with CML with monocytosis, one (50%) patient with a *BCR-ABL1* p210 driven chronic phase CML had a MO1 fraction of 98.8% and a MO3 fraction of 0.03% (Fig. 1a). Of the 43 CMML patients assessed, only 20 (46%) were newly diagnosed with de novo CMML and were treatment naïve. Of these, 15 (75%) patients had MO1 fractions >94%, with 4 (80%) of 5 flow-negative patients having *CBL* mutations and 2(40%) having concurrent autoimmune diseases leading to expansions of their MO2 fractions (Table 1). Three of five flow-negative patients had MO3 fractions <1.13% (60%). Hence, using a MO1 cut off of >94% was associated with a sensitivity of 75% and a specificity of 95.4%, while a MO3 cut off of <1.13% was associated with a sensitivity of 75% and a specificity of 82.7% (specificity calculations were carried out inclusive of cases with reactive monocytosis). When we used a dual cut off; that is a MO1 of >94%, along with a MO3 of <1.3%, the sensitivity was 60%, while the specificity was 96.5%. Of the remaining 23 (64%) CMML patients, one of five (20%) with oligo-monocytic CMML (AMC between 0.5 × 10^9^/L and1.0 × 10^9^/L), five of 10 (50%) on HMA therapy (two in a morphological CR with negative flow results), three of three (100%) on clinical trials (tipifarnib, lenzilumab, and tagraxofusp), and one of two (50%) each with therapy related CMML and post-AML revision to CMML, had MO1 fractions >94%. There was one patient with a negative flow assessment, where at the time of sample collection, criteria were met for blast transformation (Supplementary Table 2).

**Fig. 1:**
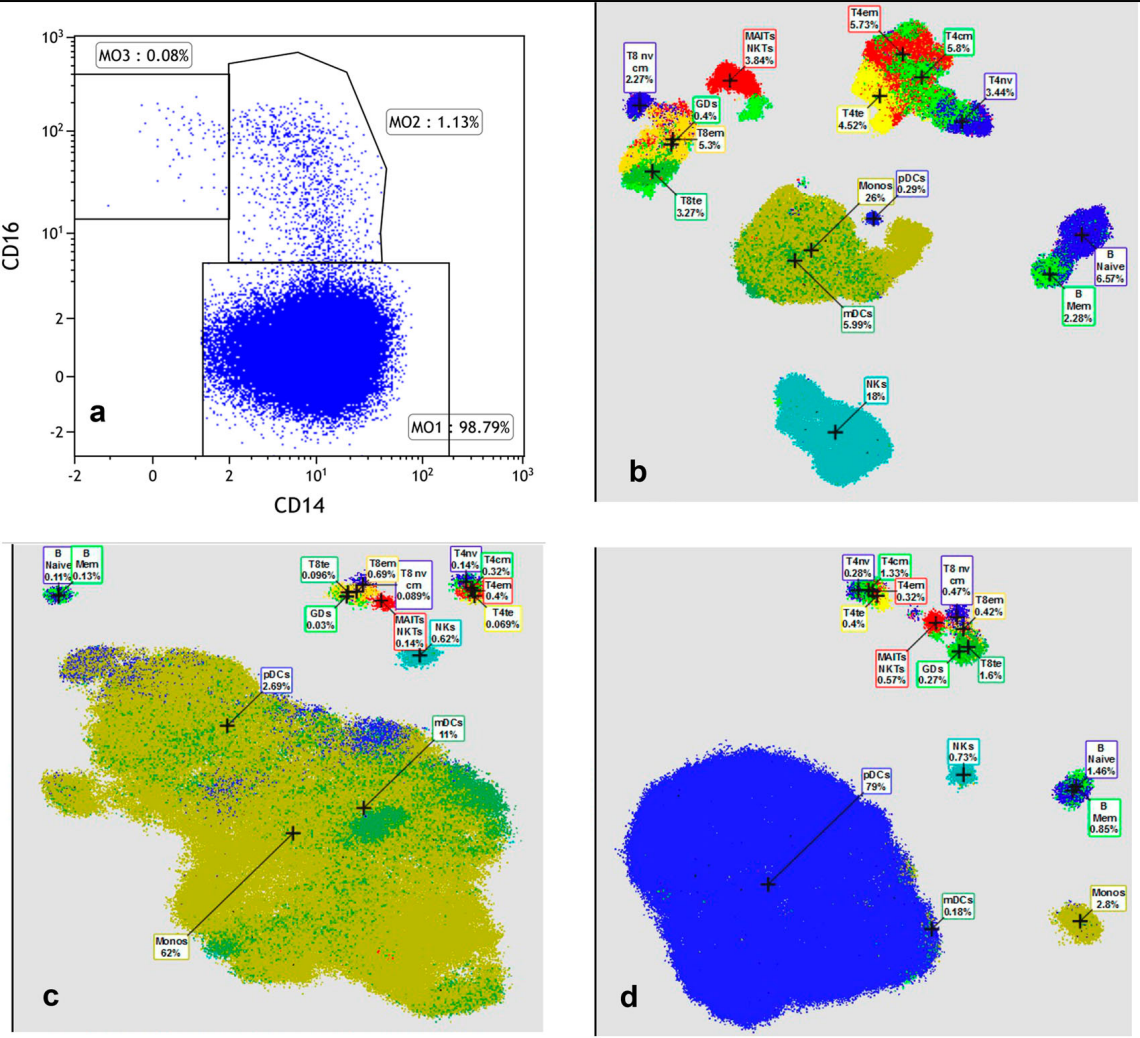
Flow cytometry and time of flight mass cytometry assessments of peripheral blood samples from patients with myeloid malignancies. **a** Monocyte subset analysis by flow cytometry on a peripheral blood sample from a patient with chronic myeloid leukemia, demonstrating an expanded classical monocyte/M01 fraction of 98% (false positive) and a M03 fraction of 0.03% (false positive). **b** Mass cytometry analysis of peripheral blood mononuclear cells from a normal healthy control demonstrating the distribution of monocytes, dendritic cells, B and T lymphocytes and NK cells. **c** Mass cytometry analysis of peripheral blood mononuclear cells from a patient with chronic myelomonocytic leukemia, demonstrating markedly expanded monocyte and myeloid derived dendritic cell subsets. Simultaneous comprehensive characterization of the immune microenvironment is also demonstrated. **d** Mass cytometry analysis of peripheral blood mononuclear cells from a patient with chronic myelomonocytic leukemia that underwent blast transformation to a blastic plasmacytoid dendritic cell neoplasm, with a markedly expanded dendritic cell pool. Note: Cell types are expressed as percentage of live events. Abbreviation: te: effector T cells, nv: naïve, em: effector memory, cm: central memory, MAIT: mucosal-associated invariant T cells; NKT: natural killer T, Mem: memory, GD: gamma-delta, mDC: myeloid dendritic cells, pDC: plasmacytoid dendritic cells, PBMC: peripheral blood mononuclear cell. Colors are qualitative only and signify different cell populations

**Table. 1:** Clinical and laboratory characteristics of treatment naïve, de novo CMML patients that underwent monocyte partitioning by flow cytometry

Sr. No	Age in years	Gender	2016 WHO diagnosis	Blood counts at the time of flow cytometry			Genotype		Monocyte partitioning on flow cytometry			Autoimmune disease
				WBC	AMC	%	Cytogenetics	Molecular genetics	MO1	MO2	MO3	
1	61	Male	CMML-1	15.3	7.3	47	46,XY [20]	*CBL, TET2, ZRSR2*	78.4	21.4	0.2	Hashimoto thyroiditis with severe hypothyroidism
2	81	Male	CMML-1	35.5	11.7	33	46,XY,del(20)(q11.2q13.3) [3]/46,XY [17]	*ASXL1, BRAF, JAK2, SRSF2, TET2*	84.6	15	0.2	No
3	78	Female	CMML-1	7.3	2.9	40	46,XX [20]	*ASXL1, CBL, TET2*	88.3	10.4	1.2	Relapsing polychondritis^a^
4	62	Female	CMML-1	18.9	3	16	46,XX [20]	*CBL, TET2*	90.7	8.2	0.9	No
5	49	Male	CMML-0	16.5	3.3	20	46,XY [20]	*ASXL1, CBL, IDH2, SRSF2*	62.3	36.2	1.4	No
6	76	Male	CMML-0	16.4	7.7	46	46,XY,del(13)(q12q22) [18]/46,XY [2]	*NRAS, SRSF2, TET2*	**94.3**	4.7	0.8	No
7	76	Male	CMML-1	19.3	4.6	24	46,XY [20]	N/A	**94.8**	3.6	1.5	No
8	72	Female	CMML-2	4.7	1.4	30	46,XX,del(5)(q31q35)[14]/47,sl, +21 [3]/46,XX [3]	None	**95.2**	4.1	0.66	No
9	80	Male	CMML-1	9	4.6	51	45,X,-Y [20]	*NRAS, SRSF2, TET2*	**95.6**	4.7	0.1	No
10	71	Male	CMML-1	12.7	3	24	46,XY [20]	N/A	**96.1**	1.8	2	No
11	78	Male	CMML-0	7.3	3.2	44	46,XY [20]	*SRSF2, TET2*	**96.5**	3.1	0.3	No
12	70	Male	CMML-1	5.9	1.5	25	46,XY [20]	*ASXL1, CBL, NRAS, TET2, U2AF1*	**97**	2.9	0.1	No
13	65	Male	CMML-0	9	1.5	17	46,XY [20]	*SRSF2, TET2*	**97.7**	1	1.2	No
14	73	Female	CMML-1	5.9	1.5	25	N/A	*TET2, PHF6*	**98**	1.8	0.1	No
15	61	Male	CMML-1	4.2	1.9	45	46,XY [20]	*SRSF2, TET2*	**98**	1.2	0.8	No
16	72	Male	CMML-1	4.1	1.5	37	46,XY [20]	N/A	**98.6**	1	0.4	No
17	65	Male	CMML-1	8.1	1.6	20	46,XY [20]	*ASXL1, BCOR, NRAS, RUNX1, SRSF2*	**98.6**	1.2	0.2	No
18	67	Male	CMML-1	15.9	1.7	11	46,XX [20]	*ASXL1, PHF6, RUNX1, SRSF2*	**98.8**	1.1	0.1	No
19	77	Male	CMML-2	32.4	10	31	46,XY [20]	*ASXL1, SRSF2, TET2*	**99.1**	0.8	0.1	No
20	83	Female	CMML-2	22.1	4.8	22	46,XX [20]	*ASXL1, BCOR, NRAS, RUNX1, SRSF2*	**99.5**	0.3	0.2	No

*CMML* chronic myelomonocytic leukemia, *WHO* World Health Organization, *WBC* white blood cells, *AMC* absolute monocyte count, *N/A* not available

^a^Patient treated with oral prednisone 10 mg daily for relapsing polychondritis

The bold values represent MO1 fractions that are >94%

While flow cytometry for monocyte subset analysis has been heralded as an important diagnostic tool for CMML, there still remain important issues, especially related to false positives and negatives. In this real world study, we assessed monocyte subsets, with cut off values of >94% for MO1 fractions and <1.13% for MO3 fractions and report a sub-optimal sensitivity for both cut off values, with acceptable specificity for a MO1 cut off >94%. In addition, we describe the first reported case of CML with monocytosis that had a MO1 fraction of >94%. Monocytosis in CML has been associated with the p190 *BCR-ABL1* isoform, and is uncommon with the p210 isoform as seen in our case^10^. Additional efforts using mass cytometry (cytometry by time of flight–CyTOF) with visual interactive stochastic neighbor embedding techniques are currently being developed by us and others, to improve the sensitivity and specificity of flow based techniques to diagnose CMML^11^. Using this method it has been shown that CD14/16 markers are adequate for detection of classical/MO1 monocytes, however, are associated with purity rates of 86 and 87% for MO2 and MO3 fractions, respectively^11^. By adding additional markers, such as CCR2, CD36, HLA-D4, and CD11c, the purity of these two fractions were increased to 98.8% and 99.1%, respectively. In addition, mass cytometry allows for the identification of fractions of plasmacytoid and myeloid dendritic cells, B and T lymphocytes and their subsets and NK cells, thus effectively profiling the immune microenvironment. In our study, in comparison to normal controls, all five CMML patients had expanded monocyte compartments along with abnormal immune subsets (Fig. 1b–d). We are currently working on studying a larger data set to assess appropriate MO1/MO3 cut off values and to asses test characteristics (sensitivity, specificity and positive predictive value). Additional markers to detect PDL1, PDL2, CTLA4, IDO1, and related immune check point regulators can also be profiled, thus adding therapeutic relevance to this assay.

In summary, we highlight some of the real world issues associated with current flow cytometry based monocyte repartitioning assays for the diagnosis of CMML. We enumerate some of the important causes of false positive and negative flow results and preliminarily describe the exciting and emerging technique of mass cytometry to help better profile monocyte subsets and comprehensively assess the immune microenvironment in this disease.

## Supplementary information

Supplementary table 1

Supplementary table 2
