# Supplementary material for: Practical limitations of monocyte subset repartitioning by multiparametric flow cytometry in chronic myelomonocytic leukemia
Source: Blood Cancer J. 2019 Aug 16;9(9):65. doi: 10.1038/s41408-019-0231-7 (PMC6697701; doi:10.1038/s41408-019-0231-7)
Supplement: Supplementary file 1 — Supplementary table 1 [file 41408_2019_231_MOESM1_ESM.pdf]

Human PBMC Phenotyping Panel for Mass Cytometry Analysis					
**No.**	**Target**	**Clone**	**Metal**	**Surface or Intracellular**	**Cell Specificity**
1	CD16	3G8	148Nd	Surface	NK Cells
2	CD8a	SK1	168Er	Surface	CD8 T-cells
3	CD20	2H7	171Yb	Surface	B-cells
4	CD4	SK3	174Yb	Surface	CD4 T-cells/Monocytes
5	CD45	HI30	089Y	Surface	Lymphocytes
6	CD19	HIB19	142Nd	Surface	B-cells
7	IgD	IA6-2	146Nd	Surface	Naïve B-cells
8	CD11c	Bu15	147Sm	Surface	monocytic DC
9	TCRgd	11F2	152Sm	Surface	Gamma/Delta T-cells
10	CD3	UCHT1	154Sm	Surface	T-cells
11	CD45RA	HI100	155Gd	Surface	Naïve T and Memory B cells
12	CD27	L128	158Gd	Surface	Memory B-cells
13	HLA-DR	L243	173Yb	Surface	monocytic DC
14	CD196/CCR6	G034E3	141Pr	Surface	Effector memory T-cells
15	CD127/IL-7Ra	A019D5	143Nd	Surface	Effector memory T-cells
16	CD38	HIT2	144Nd	Surface	Basophils
17	CD194/CCR4	L291h4	149Sm	Surface	T-regulatory cells/Th2 cells
18	CD123/IL-3R	6H6	151Eu	Surface	Plasmacytoid DC/Basophils
19	CD185/CXCR5	RF8B2	153Eu	Surface	Central Memory/Effector Memory
20	CD28	CD28.2	160Gd	Surface	Naïve CD8 T cells
21	CD183/CXCR3	G025H7	163Dy	Surface	Th1 cells
22	CD45RO	UCHL1	165Ho	Surface	Activated/Memory T-cells
23	CD24	ML5	166Er	Surface	Memory B-cells
24	CD197/CCR7	G043H7	167Er	Surface	Naïve/central memory CD8 T cells
25	CD25/IL-2R	2A3	169Tm	Surface	Activated T-cells; T-regulatory cells
26	CD14	M5E2	175Lu	Surface	Monocytes
27	CD56/NCAM	NCAM16.2	176Yb	Surface	NK Cells
28	CD66b	80H3	162Dy	Surface	Neutrophils
29	CD161	HP-3G10	164Dy	Surface	MAIT and NKT cells
