# Supplementary material for: Practical limitations of monocyte subset repartitioning by multiparametric flow cytometry in chronic myelomonocytic leukemia
Source: Blood Cancer J. 2019 Aug 16;9(9):65. doi: 10.1038/s41408-019-0231-7 (PMC6697701; doi:10.1038/s41408-019-0231-7)
Supplement: Supplementary file 2 — Supplementary table 2 [file 41408_2019_231_MOESM2_ESM.docx]

| **Supplementary Table 2: Clinical and laboratory characteristics of 23 CMML patients not included in the primary analysis** | | | | | | | | | | | | |
| --- | --- | --- | --- | --- | --- | --- | --- | --- | --- | --- | --- | --- |
| **Sr. No** | **Age in years** | **Gender** | **2016 WHO diagnosis** | **Blood counts at the time of flow cytometry** | | | **Genotype** | | **Monocyte partitioning on flow cytometry** | | |  |
|  |  |  |  | **WBC** | **AMC** | **%** | **Cytogenetic** | **Molecular** | **MO1** | **MO2** | **MO3** |  |
| 1 | 67 | Male | CMML-1 | 31 | 4 | 12.9 | 46,XY,t(3;12;6)(p21;q21;q23) [8]/46, XY [12]. | *ASXL1, EZH2, TET2* | **98.5** | 1.4 | 0.1 | On lenzilumab (KB003) therapy |
| 2 | 38 | Male | CMML-1 | 3.8 | 0.6 | 15.7 | 46,XY [20] | *NA* | 92.4 | 4 | 3.6 | AMC <1 x 10^9^/L |
| 3 | 70 | Male | CMML-0 | 4 | 0.7 | 17.5 | 46,XY [20] | *PHF6, RUNX1, TET2, U2AF1, ZRSR2* | **94.5** | 5.3 | 0.2 | On Tagraxofusp (SL-401) therapy |
| 4 | 72 | Male | CMML-2 | 42.2 | 8 | 18.9 | 47,XY,+21 [20] | NA | **96** | 3.8 | 0.2 | Post AML revision back to CMML |
| 5 | 68 | Male | CMML-0 | 7.1 | 0.9 | 12.7 | 46,XY [10] | *ASXL1, CBL, RUNX1, U2AF1* | 85.9 | 13.8 | 0.3 | AMC <1 x 10^9^/L |
| 6 | 78 | Male | CMML-0 | 6.7 | 0.9 | 13.4 | 46,XY [20] | *ASXL1, TET2, NRAS* | 93.4 | 2.8 | 3.7 | On Azacitidine therapy |
| 7 | 75 | Male | CMML-0 | 4.6 | 0.8 | 17 | 46,XY [20] | *SRSF2, TET2* | 92 | 7.9 | 0.1 | AMC <1 x 10^9^/L |
| 8 | 78 | Male | CMML-0 | 4.6 | 0.5 | 10.8 | 46,XY [20] | *SRSF2, TET2* | 86.8 | 12.2 | 1 | AMC <1 x 10^9^/L |
| 9 | 63 | Female | CMML-2 | 2.3 | 0.5 | 21.7 | 45,XX,add(1)(p32),psu dic(5;6)(q11.2;q13),-7,der(12;13)(q10;q10),+2mar [20] | *DNMT3A, PTPN11, TET2, TP53* | **98.4** | 1.5 | 0.1 | AMC <1 x 10^9^/L |
| 10 | 68 | Female | CMML-1 | 14.8 | 2.5 | 16.8 | 46,XX,del(20)(q11.2q13.1) [17]/46,XX [3] | *ASXL1, NRAS, TET2* | **95.3** | 4.5 | 0.1 | On Decitabine therapy |
| 11 | 70 | Female | CMML-1 | 124 | 16.1 | 12.9 | 46,XX [20] | *JAK2, SRSF2, TET2* | **98.5** | 1.1 | 0.4 | On Azacitidine therapy |
| 12 | 65 | Male | CMML-2 | 27 | 6.9 | 25.5 | 46,XY [20] | *ASXL1, RUNX1, WT1* | **99.6** | 0.4 | 0 | On Decitabine therapy |
| 13 | 61 | Female | CMML-0 | 1.6 | 0.1 | 6.2 | 46,XX [20] | *CBL, IDH2, NRAS, SRSF2* | 46.4 | 31.8 | 21.8 | On Decitabine therapy |
| 14 | 66 | Male | CMML-1 | 70.7 | 45.3 | 64 | 46,XY [20] | *ASXL1, IDH2, SRSF2* | 56.5 | 43.2 | 0.3 | Post CMML leukemic transformation at the time of blood draw |
| 15 | 66 | Male | CMML-0 | 41.9 | 1.3 | 3.1 | 46,XY [20] | *ASXL1, NRAS, TET2, SRSF2* | **95.6** | 4.1 | 0.3 | On Tipifarnib therapy |
| 16 | 70 | Male | CMML-0 | 17.7 | 1.6 | 9 | 46,XY [20] | NA | 71 | 28.4 | 0.6 | On Azacitidine therapy |
| 17 | 70 | Male | CMML-1 | 3 | 0.5 | 16.6 | 46,XY [20] | *ASXL1, EZH2, BCOR, SF3B1* | **95.8** | 2.9 | 1.3 | On Azacitidine therapy |
| 18 | 63 | Male | CMML-0 | 48.3 | 12.6 | 26 | 46,XY [20] | *CBL, SRSF2, TET2* | **95.9** | 4.2 | 0 | On Decitabine therapy |
| 19 | 72 | Male | CMML-0 | 29.1 | 4.4 | 15.1 | 46,XY [20] | *SETBP1, CBL, TET2* | 85.3 | 11.4 | 3.3 | Therapy related CMML |
| 20 | 70 | Male | CMML-0 | 9.3 | 1.4 | 15 | 46,XY [20] | *ASXL1, KIT, U2AF1* | **94.7** | 5.3 | 0 | Therapy related CMML |
| 21 | 56 | Male | CMML-1 | 2.9 | 0.1 | 3.4 | 46,XY [20] | *IDH1, SRSF2, TET2, ASXL1, CBL* | 87.8 | 11.2 | 1 | On Azacitidine therapy |
| 22 | 69 | Female | CMML-2 | 14.7 | 3.1 | 21 | 46,XX [20] | NA | 86.5 | 13.8 | 0.7 | On Azacitidine therapy |
| 23 | 73 | Male | CMML-0 | 28 | 7.8 | 27.8 | 46,XY [20] | *ASXL1, EZH2, JAK2, RUNX1, SRSF2* | 83.7 | 16 | 0.3 | Post AML revision back to CMML |

Key: CMML: Chronic myelomonocytic leukemia; WHO: World Health Organization; WBC: White blood cells; AMC: Absolute monocyte count: AML: Acute myeloid leukemia; N/A: not available
